# Feasibility and Usability of Low-Field Magnetic Resonance Imaging for Pediatric Neuroimaging in Low- and Middle-Income Countries: A Qualitative Study

**DOI:** 10.2147/MDER.S478864

**Published:** 2025-02-14

**Authors:** Erin Rowand, Rosemond Owusu, Alexandra Sibole, Elizabeth Abu-Haydar, Jaclyn R Delarosa

**Affiliations:** 1 Medical Devices and Health Technologies, PATH, Seattle, WA, USA

**Keywords:** infant neuroimaging, portable MRI, human-centered design

## Abstract

**Background:**

The burden of neurological disorders in low- and middle-income countries (LMICs) may be underestimated due to the limited number of diagnostic imaging devices and trained specialists to operate and interpret scans. Recent advancements in low-field (<100 milliteslas) magnetic resonance imaging (LFMRI) hold significant promise for improving access to pediatric neuroimaging due to the technology’s lower costs, portability, and reduced infrastructure and training requirements.

**Purpose:**

Explore user needs and experiences on the training and use of a portable LFMRI for pediatric neuroimaging in LMICs.

**Methods:**

We conducted qualitative interviews with end users of the LFMRI systems across 11 sites in Bangladesh, Ethiopia, Ghana, Malawi, Pakistan, South Africa, Uganda, and Zambia. A semi-structured questionnaire with open-ended questions on usability and feasibility was used to encourage participants to share their experiences and opinions on ease of use, user satisfaction, and integration into local health systems.

**Results:**

Among 46 participants, key challenges were reported in infant positioning, power stability, and internet connectivity. Suggestions included developing reference materials with content and format tailored to local needs and contexts, conducting refresher trainings, and providing education that includes technical and maintenance support crucial for appropriate utilization and implementation sustainability.

**Conclusion:**

This study underscores the importance of incorporating human-centered design principles and user feedback into identifying and resolving usability issues, sharing insights for successful integration of LFMRI within existing health care infrastructures in LMICs, and optimizing LFMRI use for pediatric populations.

## Introduction

Estimates reveal 52.9 million children under 5 years old have developmental disabilities worldwide, with a staggering 95% of them residing in low- and middle-income countries (LMICs).^1^ The disproportionate number of children affected by neurological conditions in LMICs is a critical public health concern, highlighting the need for greater focus on prevention, diagnosis, early intervention, and management strategies to improve neurological health outcomes.

Magnetic resonance imaging (MRI) is a fundamental, noninvasive imaging tool for examining brain structure and understanding brain health. Yet, an estimated two-thirds of the world does not have access to MRI.^2^ Limited access to neuroimaging remains a key barrier to identifying, treating, and monitoring brain injury and neurodevelopment in the early postnatal years (from birth to 5 years of age)—a critical period in brain growth and development.^3^ The availability of traditional MRI systems in LMIC is influenced by several key factors, including the significant costs associated with acquisition and maintenance, inadequate infrastructure, and a lack of trained personnel.^4^,^5^

Use of new, less expensive ultra-low-field (less than 100 milliteslas) magnetic resonance imaging (LFMRI) technologies for neuroimaging and other whole-body applications is a nascent and growing research area in both high-income countries and LMICs.^6–12^ Advances in LFMRI have resulted in point-of-care scanners that use a lower magnetic field, thus offering a safer, smaller, quieter, and more flexible alternative for infants and young children than conventional high-field MRI. Portable LFMRI is a novel system with reduced power requirements and a compact design that is currently being assessed for various clinical applications, including neuroimaging at the patient’s bedside in intensive care unit (ICU) settings.^13–15^ The feasibility of LFMRI is also being actively investigated for various pediatric imaging applications, including potential uses in neonatology and studies on nutrition and brain development.^7^,^14^ The benefits of portability, reduced training requirements, and adaptability make LFMRI a valuable tool in both advanced medical settings and resource-limited environments. Furthermore, ongoing research aims to implement standard clinical neuroimaging protocols and innovative reconstruction techniques using machine learning to enhance image quality and processing speed.^16–18^ The Ultra-low-field Neuroimaging In The Young (UNITY) initiative, a global collaborative effort to deliver improved tools for measuring infant neurodevelopment, has provided a low-cost LFMRI scanner (Hyperfine’s Swoop^®^ Portable MR Imaging^®^ System) to more than 20 hospital and clinical research sites across the world to date.^18^,^19^ We conducted a usability evaluation of the training and use of LFMRI for pediatric neuroimaging research in LMICs in 11 locations across Africa and Asia to receive the device as part of the UNITY project launch. Assessing how easily LFMRI can be used and integrated into existing healthcare systems is particularly important in LMICs, where staff have varying levels of training and experience and operate in diverse environments. Our aim was to collect user insights that not only could help developers and policymakers understand the needs, concerns, and preferences around the design and operation of LFMRI in real-world settings and pediatric populations, but could also guide decisions on resource allocation, infrastructure development, and educational resources to build the capacity of LMIC professionals in neuroimaging to support better diagnosis, treatment, and research in neurological diseases and disorders and ultimately improve brain and neurological health outcomes in LMICs.

## Methods

### Study Design

We conducted a qualitative study to better understand the context of use, potential barriers to use, and user perceptions of the training and LFMRI system in LMICs. Our descriptive approach explored challenges and potential solutions for improving training and infant positioning methods, and assessed perceptions of ease of use, user satisfaction, and the feasibility of integration into local health systems of LFMRI among users for pediatric neuroimaging in LMIC settings.

The study was undertaken at 11 sites that received Swoop^®^ LFMRI scanners and training as part of the larger UNITY project. Usability measures included ease of use, training efficiency, user satisfaction with the LFMRI system for pediatric image acquisition, and desired changes to training, scanner design, and troubleshooting support that would improve the ability of users to obtain images from children. Feasibility questions considered installation of the scanner into the users’ specific environment, workflow efficiency and potential clinical applications of LFMRI, interactions with parents and caregivers, and other systemic factors such as power infrastructure affecting optimal use of the scanner.

Participants and the public were not involved in the planning, design, or conduct of this research, nor the reporting of the results.

### Materials

**Figure 1:**
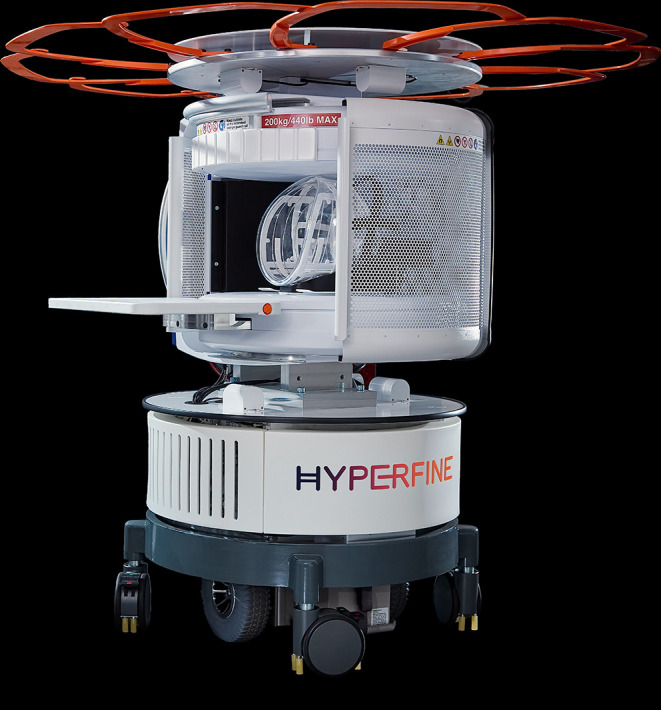
Swoop^®^ Portable MR Imaging^®^ System. Image courtesy of Hyperfine.

The Swoop^®^ LFMRI scanner’s head coil is made of a solid, clear plastic designed to hold the subject’s head still during the scan. The coil is surrounded by a perforated scanning cage with multiple access doors that allow for positioning (Figure 1). The scanner comes with a detachable iPad tablet with proprietary software that allows providers to tailor the scanning protocol appropriately and then perform the scan. The mobility of the scanner is powered by an internal battery that is charged while being plugged in via the power cord.

In-person training was provided by either the manufacturer or another experienced LFMRI user, with training content including a didactic lecture and presentation that covered basic functions of the machine, safety practices, and the instructions for use. This was followed by hands-on sessions during which participants practiced handling the infant positioner and using the iPad and the scanning software to scan each other, as well as maneuvering the scanner.

### Study Setting and Participant Recruitment

A purposive sample was drawn from all users who received training from the manufacturer or UNITY consortium partner between April 2021 and June 2024 in the 11 study sites with a Swoop^®^ LFMRI scanner. Participants were recruited from the pool of trained operators in Bangladesh, Ethiopia, Ghana, Malawi, Pakistan, South Africa, Uganda, and Zambia to gather a variety of perspectives on the usability and feasibility of the Swoop^®^. Trained users aged 18 or older working in a role that required them to operate or directly interact with the scanner or supervise its use at the LMIC site and willing and able to provide informed consent were eligible. All potential participants were contacted by Email to voluntarily participate in the study and assured of their right to decline or withdraw at any point, and all who accepted the invitation to participate were interviewed. A copy of the verbal consent document was electronically shared with participants during recruitment, and their verbal consent to enroll in the study was recorded using the meeting software (Microsoft Teams) by investigators prior to the start of the interviews.

### Data Collection and Analysis

We conducted two rounds of post-training interviews that contained participants from a single site with no more than five participants per session. The initial interviews were conducted within one to three months after users completed training and focused on installation, setup, and training. The second interviews were held four to six months later after users had completed a minimum of twenty scans for their study and delved into usability and feasibility. All interviews were conducted remotely after obtaining verbal informed consent and followed a semi-structured interview topic guide in English (supplementary material). Each interview lasted from 30 minutes to one hour.

The interviews were audio-recorded using the meeting software (Microsoft Teams) and transcribed into an electronic database where the data were cleaned, coded, de-identified, and analyzed by manually sorting and summarizing emerging themes using descriptive statistics and a qualitative codebook employing emergent themes drawn from the data. Three different researchers performed the transcription and coding of the data and maintained alignment on codes using a codebook. We maintained confidentiality by ensuring no identifying information was included in the analysis. The transcripts or recordings were not shared with the participants after the interviews.

## Results

Across 11 sites in eight LMICs, 46 end users consisting of research and clinical experts who received training on the LFMRI system were interviewed remotely between December 2021 and May 2024. Participants represented a mix of medical and clinical, radiology, research, and technical staff (Table 1). Of the 12 medical and clinical staff interviewed, seven were nurses, three were physicians, one was a neuropsychologist, and one was a medical officer. Of the 15 radiology staff interviewed, in addition to a radiologist and a manager, six were imaging technicians, four were radiographers, and three were sonographers. The 14 research staff included four principal investigators, one assistant, six study coordinators, and three study staff. The five technical staff consisted of two information technology staff and three biomedical engineers. At least one participant per site had prior experience with high-field MRI.

**Table 1:** Distribution of Study Participants From Each of the Sites and Their Primary Roles

Country	Location	Site	Facility type	Participant Sample Size	Participant role			
					Medical and Clinical Staff	Radiology Staff	Technical Staff	Research Staff
Pakistan	Karachi	Aga Khan Hospital	Private tertiary hospital	6	0	3	0	3
South Africa	Cape Town	Cross University Brain Imaging Center	Public medical imaging facility	2	0	0	0	2
	Pretoria	Kalafong Hospital	Public tertiary hospital	2	1	1	0	0
	Johannesburg	Chris Hani Baragwanath Hospital	Public tertiary hospital					
Uganda	Kampala	Kawempe National Referral Hospital	Public tertiary hospital	4	1	2	0	1
Zambia	Lusaka	University Teaching Hospital	Public tertiary hospital	5	1	3	0	1
Malawi	Zomba	Zomba Central Hospital	Public tertiary hospital	12	6	2	0	4
Ethiopia	Bahir Dar	Felege Hiwot Regional Referral Hospital	Public tertiary hospital	2	1	1	0	0
Ghana	Accra	Korle-Bu Teaching Hospital	Public tertiary hospital	9	1	3	4	1

Users reported their experiences using the LFMRI scanner with children aged 0 to 11 years, with most of the infants scanned being under 6 months old. Interviews with participants revealed major subthemes around usability and feasibility of LFMRI implementation for pediatric neuroimaging research and potential clinical applications (Table 2).

**Table 2:** Summary of Major Subthemes and User Feedback

Theme	Facilitators	Desired improvements
**Usability**		
**Perception and acceptability of training**	In-person, hands-on practice time scanning infants and children	More detailed instruction on scanning protocols
	User-led, peer-to-peer, decentralized training by users from other LMIC settings	Short refresher trainings and education materials to support peer training
	Minimized time between training completion and when users start scanning	Pictorial references and quick guides
	Inclusion of biomedical engineers in initial trainings to equip them to assist with troubleshooting, maintenance, and repair	Education on common problems and how to solve them
	Availability of troubleshooting support before and after initial training	General best practices for cleaning
**Pediatric image acquisition**	Use of positioning aids to keep children immobile and asleep	Expanded adjustment of machine settings
	Creating a welcoming environment (noise, lighting, temperature) to keep the children comfortable and asleep during the scan	Faster acquisition techniques to minimize the time children need to keep still
		Options to minimize or mask the scanner noise
**Ease of use and satisfaction**	Small footprint of the machine	Greater cushioning and ventilation
	Interface simple and easy to use	Larger monitors, zoom functionality, or print options to improve image accessibility
	Easy-to-use scanner design required minimal initial training	Optimized comfort for a range of children’s ages and sizes
		Increased ventilation inside scanning compartment
**Feasibility**		
**Technology installation and troubleshooting**	Careful planning and preparation of the facility prior to installation of the scanner	Greater strength and stability of mobile hotspot connections or reliable alternatives
**System integration**	Dedicated and stable power supply and internet connectivity	Capability of built-in battery to provide backup power to avoid disruptions during scanning
	Cloud-based platforms for remote access, software upgrades, and data sharing.	Increased internal data storage capacity to prevent overwriting during connectivity disruptions
	Network optimization to reduce bandwidth consumption and improve data transmission	
**Workflow efficiency and potential clinical applications**	Easy-to-use features facilitating flexible maneuvering for scanning at a child’s bedside	Reduced weight and improved movement speed to expand mobility to other areas of the facility
	Adequate battery range for transport across the facility	Capability to scan other parts of the body for broader clinical applications
	Several advantages over high-field MRI to improve workflow efficiency	
	Prospect of reducing the load on their traditional MRI scanner	
**Preparation and education of parents and caregivers**	Communication and cooperation with the parents and caregivers of children	Placards or posters of children in the scanner to display
	Explanations of the scanning procedure in the local language	

### Usability

#### Perception and Acceptability of Training

Users at all sites reported that the scanner was easy to train for use and that they were able to perform scans after the training. Seven sites were able to initiate recruitment and practice scanning infants and young children during the training. All sites reported they used their knowledge to train others who were not present at the original training so that use of the machine could be expanded. Recommendations the users had to improve the training were more time to practice scanning infants and children while the trainers were present, more detailed instruction on scanning protocols, and less time elapsed between the training and start of the neuroimaging study or other clinical use of the machine.

**Figure 2:**
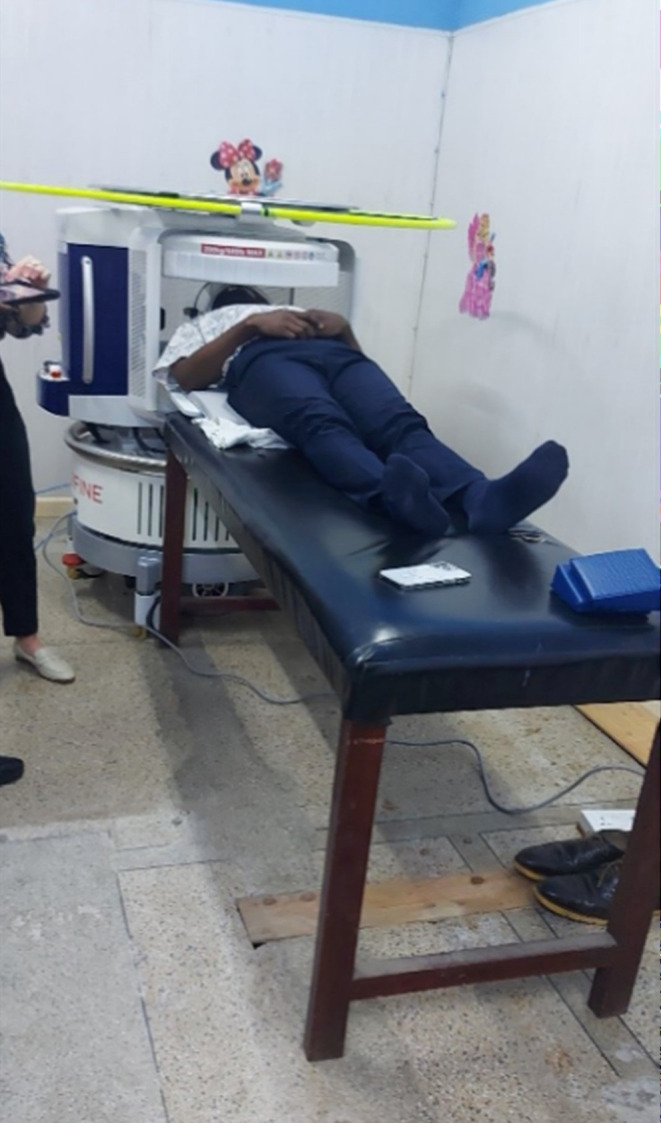
Study participant laying in scanner during training.

The sites in Ethiopia and Ghana that were trained by South Africa–based users of the system shared that the user-led, peer-to-peer training by experienced users training others in contextually similar settings was an effective approach for providing MRI education and a particularly valuable tool for facilitating knowledge sharing and skill development (Figure 2). The trainees who received the decentralized peer-training model reported that these trainings facilitated direct observation of MRI scans, interactive case discussions, and collaborative peer-to-peer learning which imparted practical knowledge and a deeper contextual understanding of the scanner. One of the operators in South Africa who conducted peer training for the site in Ghana said, “It was good to go to the site and work with the team there to help adapt strategies that work for their local context”.

Online training and troubleshooting sessions were also provided to the sites, both before and after the in-person training, however, hands-on training was reported by users at all sites to be the most preferred way to learn how to use the scanner. Training topics most commonly requested by participants included troubleshooting of common problems and how to solve them, best cleaning practices for the machine, and more time to practice on infants and children.

Participants in Malawi and Zambia suggested that pictorial references and a quick guide would help users remember how to use the scanner, especially when considering delays between training and use of the equipment, and in the event of staff turnover. Furthermore, short refresher trainings, videos, and/or online modules would support a peer training approach that trained individuals could employ to train additional providers in use of the scanner, which would also alleviate the need for a training team to revisit a location. Site-local biomedical engineers requested they be included in trainings to ensure their knowledge of the machine would be adequate for technical troubleshooting, maintenance, and repair.

#### Pediatric Image Acquisition

Most children scanned were under 6 months old. Methods for preparing infants for scanning were shared across sites and included swaddling, mothers breastfeeding them beforehand, or administration of the naturally occurring hormone melatonin to effect sleep. Once asleep, infants were placed in the scanner using the site’s chosen positioning method, and the scan would begin after the scanner’s localization and alignment step.

**Figure 3:**
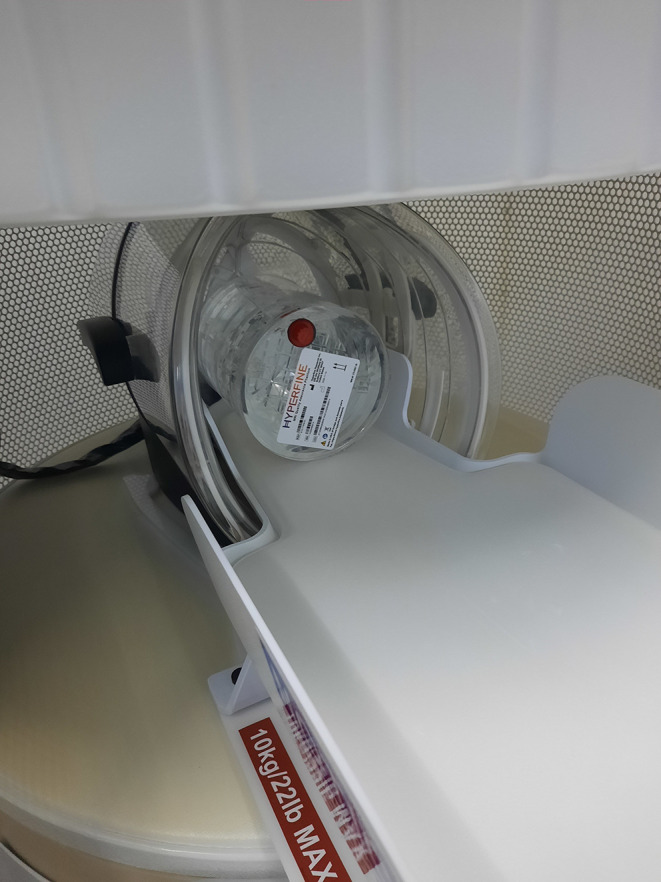
Calibration phantom.

Methods to position the children varied greatly by site, based on availability of positioning aids and scanning workflows unique to each site. An infant positioner tray meant to facilitate positioning of pediatric subjects came with the LFMRI system (Figure 3), but users in only two sites reported using it consistently due to challenges with the design, such as (1) inadequate cushioning and ventilation and environmental factors (noise, lighting, temperature) that contributed to infants waking during scans; and (2) ranges in children’s ages and sizes, which rendered the tray ineffective for many subjects. Children who were too large for the infant positioner tray laid on a bed or table positioned next to the scanner.

**Figure 4:**
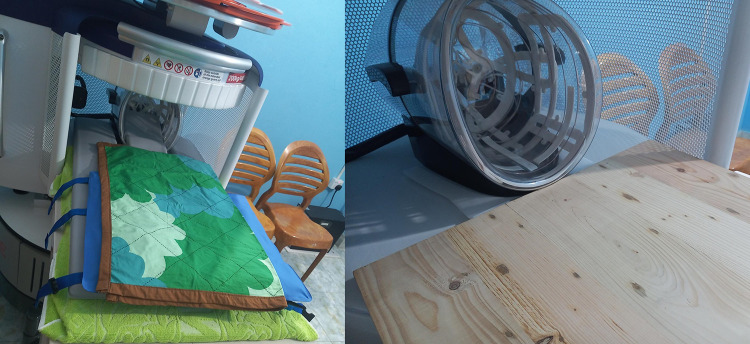
Wooden plank configuration used in Bangladesh.

Participants at multiple sites encountered problems with the children’s chins and heads tilting forward due to a gap between the floor of the scanning cage and the head coil, causing the images to be incomplete. A site in Bangladesh made their own wooden positioning aid to eliminate this gap to prevent forward head tilting and improve image quality (Figure 4).

Scanning children under three years old proved to be one of the biggest challenges across all sites since they needed to sleep for 30 to 45 minutes in the scanner, on average, to remain still enough for a good-quality image to be generated. Providing calming music eased anxiety and helped the children older than eight years old remain comfortable and still during the scan.

Motion challenges resulted in poor image quality, and scanning times were shortened due to infant discomfort and waking. Many sites improved comfort and security by cushioning the tray with blankets or foam padding, and others utilized an infant immobilizer to securely keep infants asleep for longer, enhancing scan completion rates.

The loud beeping sound emitted by the scanner awakened infants before imaging, requiring soothing by caregivers to facilitate the procedure. Various methods such as using cotton balls, foam padding, or blankets were employed to protect infants’ ears from the noise, with most sites adapting padding to cover both ears and secure the position of the head. Requests for noise reduction or local adjustment options were common among sites to improve the children’s comfort during scans.

#### Ease of Use and Satisfaction

Due to the solid design of the head coil, users reported that ventilation in the cage was insufficient. The head coil was also reported as too small for masked and intubated patients, and its location was not ideal for all children. The location of the head coil is slightly elevated from the scanning cage floor and changed the orientation of the head inside the coil for many children, causing parts of the brain to be cut off from the image. As a nurse in Malawi said,

Main issue is babies waking up due to overheating. The tray raises the baby’s head too high and causes less ventilation. There is no space in the coil to help with [airflow].

The iPad was seen as easy to misplace since it is not attached to the scanner. Many sites indicated they would prefer to have a large monitor for interfacing with the machine. Furthermore, the field-of-view size in the program was not precise enough or easily adjustable.

Overall, however, users seemed satisfied with the functions the scanner provided and were impressed with the quality of the images when considering the footprint of the machine and thought the lower-quality images were still very useful. As a participant in Zambia said,

The interface was easy to use and not complicated, the machine was much easier to use than traditional MRI.

Suggested improvements included the ability to pause the scan to give users more time to make positioning adjustments or attend to interruptions and the ability to edit and print the scans from the tablet to improve usability and image accessibility.

### Feasibility

#### Technology Installation and Troubleshooting

Overall, users in all sites were successful at uncrating and installing the LFMRI system, and all study locations met the requirements for housing the device, sometimes with minor remodeling. Many doors were too narrow for the scanner to fit through, and a handful of sites remodeled a designated room for the machine with a door large enough to accommodate the scanner. During facility preparation, it was important to ensure there were no tipping risks, inclines, or floor gaps outside of what the machine is rated for to prevent injury to personnel and damage to the scanner during maneuvering. Adequate room space to accommodate parents and caregivers comfortably was highly valued by participants. Ensuring the scanner was located in a quiet, private space was important so mothers who needed to breastfeed to help their child to sleep for the scan could do so comfortably. As a participant in Ghana said,

It is comforting for the mothers to be in the room with the babies and to see the images after. Even though they don’t really understand the scans they still enjoy seeing them.

While most sites (7 of 11) did not experience connectivity problems during installation, two sites required extra assistance when setting up their machine and two sites experienced persistent internet barriers. A site in South Africa reported intermittent disconnections between the scanner and iPad and were unable to resolve the problem on their own despite numerous troubleshooting efforts. The problem was solved when a team from Hyperfine investigated and fixed the issue. The users in Pakistan also reported connectivity problems when setting up their machine, however trainers were able to resolve them before leaving the site. Users in Pakistan also reported that post-training conference calls were helpful for continued support regarding software updates. Users in Zambia and Uganda reported connectivity issues related to poor internet quality and disconnections from the Swoop^®^ hotspot. Connecting the scanner to the ethernet was desired to fix this problem, however this would not allow them to use the scanner portably.

#### System Integration

In all sites, power outages and electrical fluctuations were common root causes of many connectivity problems the local teams encountered. Inconsistent connectivity was a routine bottleneck for data transfer and processing, as well as software upgrades. Some users noted that it would be helpful if the built-in battery provided backup power to avoid disruptions during scanning and potential data loss during sudden power interruptions. If there was a need to restart the scanning process, it was frustrating for both health care providers and caregivers of the children and lead to longer scanning sessions. The analog-to-digital circuit boards in the LFMRI systems in two sites in Bangladesh were severely damaged by voltage fluctuations. With training from the manufacturer and by following the user manual, biomedical engineers for the sites replaced the circuit boards. Following these incidents, both sites utilized uninterruptible power supply systems to protect their scanners from voltage fluctuations and overcome recurring and unscheduled blackouts. A generator was used when there was a higher proportion of outages; however, this was not desirable due to the generator noise.

Users commented that the iPad had a limited amount of storage space for scans; and therefore, wireless uploads had to be completed regularly so that data on the tablet were not lost. When power or connectivity was lacking, scans could not be uploaded to the server. As a participant in Pakistan said,

Transferring info from the scanner to the [Picture Archiving and Communication System (PACS)] can take a while depending on connectivity but this is a network issue and not a device issue.

Overall, reduced bandwidth requirements and more data storage solutions could benefit sites with less reliable internet connectivity.

#### Workflow Efficiency and Potential Clinical Applications

Users expressed enthusiasm for the scanner’s portability, seeing potential to enhance MRI access for immobile patients, with easy-to-use features facilitating flexible maneuvering and storage. As a participant in Pakistan said, “The portability of the scanner is going to change the way we practice medicine. We have been able to use the machine all over the hospital”. Participants from one-third of the site locations reported mobile use of the LFMRI system for scanning at a child’s bedside.

However, the scanner was stationary in most facilities (8 of 11), with weight restrictions, lack of space to maneuver, and inability to fit through standard door widths cited as the most common reasons. Battery range was adequate for moving the LFMRI device throughout hospitals and clinics; however, slow movement speed and challenging location adjustments could impact emergency care and expansion of usage scenarios to operating rooms.

When users in Pakistan reported to Hyperfine their scanner’s battery not withstanding longer transit distances, a battery replacement process ensued. Within one week they received a new battery and were able to install it using the user manual and an online training with Hyperfine engineers. The time needed for battery replacement from initial problem diagnosis to restoration of full machine function was ten days.

Users noted multiple improvements over high-field MRI, such as increased scanning speed, lower scanning noise, more accessible subject positioning, faster image acquisition and availability, and fewer requirements for machine climate control. While impressed with the image quality of the images, users indicated that the machine would have more value if it could scan other parts of the body, such as an arm or leg, and not just the head.

Beyond the scanner’s use in the UNITY research studies, many users suggested the prospect of other clinical scenarios of use: in ICUs, including neonatal ICUs; to alleviate the patient load on their conventional MRI scanners; and for pre- and post-operative imaging, to name a few. As a participant in Malawi said,

The scanner would be useful in ICUs for both infants and adults, and specifically helpful for meningitis patients.

A participant in Ghana also said, “The scanner has reduced the load on the traditional MRI scanner”.

#### Preparation and Education of Parents and Caregivers

Many participants provided a verbal explanation of the scanning procedure in the local language to those who were scanned or had their children scanned. Participants in Malawi, Ethiopia, South Africa, and Ghana reported fear of the scanner from parents and caregivers unfamiliar with MRI, and the providers suggested culturally sensitive placards or posters displaying infants in the scanner could help ease worries. Caregiver involvement was crucial in helping prepare children for the MRI scan and providing hands-on or emotional support to keep children calm and comfortable during the imaging procedure. A participant in South Africa said,

We love that it is very interactive and inclusive of the mothers – they can be in the room and see the scans and images popping up on the tablet.

## Discussion

This qualitative human-centered design study on the use of LFMRI for pediatric neuroimaging revealed myriad opportunities to enhance the usability experience in LMICs, considering local resource and workforce constraints that can hinder successful implementation. Engaging end users throughout the deployment process not only ensures LFMRI technology is tailored to the local needs and specific challenges of LMICs, but also fosters a sense of ownership that can lead to higher acceptance and the development of strategies to support effective and sustainable integration.^7^,^8^,^20^,^21^ Studies evaluating the use of LFMRI technology have focused on improving imaging capabilities in LMIC and addressing specific health challenges prevalent in these regions.^17^,^22^,^23^ In recent years, there has been an increasing effort to assess the diagnostic efficacy of LFMRI.^2^,^9^,^24^,^25^ Studies exploring implementation of LFMRI in healthcare environments within LMIC and the unique challenges and solutions from the perspectives of stakeholders in Malawi and Uganda have noted the importance of community engagement in planning, training, and capacity building.^10^,^26^ This study emphasizes the significance of local input and provides user insights, concentrating on shared and critical considerations regarding training and utilization to enhance LFMRI accessibility for pediatric neuroimaging across a variety of geographical areas, nations, and cultural landscapes.

Training that contributed to successful learning outcomes emphasized a hands-on approach and culturally appropriate, user-led sessions tailored to specific local needs and circumstances. Unique to the experience of users in this study was the later use of decentralized peer training models involving collaboration with other experienced users also from LMICs. In-person training by users with existing LFMRI expertise who were conducting similar studies in neighboring countries was an essential and effective component for successful introductions. Notably, users expressed appreciation for the benefits and value of cascade training as a sustainable approach to knowledge transfer and continuity, promoting a sense of ownership, and devising locally appropriate solutions. Opportunities to optimize learning in LMICs include developing a standardized LFMRI training curriculum and resources specific to the needs of LMIC settings for trainers and establishing online and on-site mentorship and support systems beyond the initial training period.^27^

Participants in this study shared insights on their difficulties with head positioning of young children within the adult-sized coil, as previously described by other users.^8^ Flexible positioning aids and easily adjustable settings such as indicator volume could help to increase the overall usability of the LFMRI device for children. User feedback revealed desire for modifications to the scanner design to improve ventilation and better accommodate pediatric subjects. Maximizing hands-on trainee practice time with children, identifying age-appropriate preparation techniques and accessories to ensure the correct position and comfort of the child, providing visual aids for enhancing memory and recall on proper positioning and detection, and reducing motion artifacts could help to ensure the consistency and efficiency of good-quality image acquisition.

The significant challenge of keeping non-sedated children immobile or not waking them during the LFMRI scan may be improved by methods to minimize disturbances, optimize the scanning environment, provide distraction activities for older children, and continue to advance artificial intelligence and fast imaging protocols.^28^,^29^ Consideration should also be given for any infant handling and immobilization methods applied prior to scanning and their compatibility with medical support equipment that may be needed during the scan, such as mechanical ventilation, pulse oximetry, and temperature and heart rate monitoring.^14^

Infrastructure challenges, particularly related to connectivity, data transfer, and the dependency on internet access (software functionalities and updates, cloud-based storage), hindered the accessibility and effectiveness of LFMRI in the study countries. Additionally, internet quality impacted the ability of users to access online educational and training resources or participate in remote consultations.

Possible solutions include ensuring the imaging system is adapted to accommodate power outages, low bandwidth, and poor internet speeds, or providing another means for implementing a local data storage backup system on a secure drive to prevent data loss.^2^ Strategies to effectively meet connection and bandwidth requirements include advanced MRI techniques in development to reduce the size of imaging data and increase the speed of data transfer.^30^ Implementing offline solutions, such as offline data analysis and visualization software, and maintaining a library of offline educational materials like user manuals and video tutorials, should be prioritized to help overcome connectivity limitations, especially in settings where power outages are frequent.

The technical expertise, training requirements, and resources needed to operate and maintain the internet-based components and software used for data acquisition, analysis, image processing, security, and storage are additional points to be considered. Equipping network and information technology staff with the knowledge to troubleshoot common software errors or technical problems during data acquisition is as crucial as training users on the LFMRI hardware components, their functions, and proper operation. Collaboration and knowledge-sharing between LMIC institutions and research centers to share best practices and apply creative solutions could be a valuable resource toward advancing progress on digitizing radiology and capacity development in radiology information systems and data management.

Importantly, unstable power infrastructure was noted in our study to be a major root cause of unreliable internet connection. In terms of usability, this not only led to disruptions in data acquisition but could lead to damage to the LFMRI or other devices, like routers or modems. The significantly reduced power requirements of portable LFMRI compared to high-field MRI systems permits further exploration into sustainable facility power supply systems and could enable a rechargeable, built-in, or portable battery supply to ensure optimal performance and lifespan of the device is increased. Small investments in built-in or external voltage protection measures or uninterruptible power supply devices could safeguard delicate device components against harmful voltage fluctuations, even in areas with stable power. These considerations are important when designing a system for use in LMICs to address the bottleneck of medical device longevity in LMICs.^31^

This study sheds light on the potential additional scenarios for LFMRI use. Its positive impact on workflow and feasibility in LMICs demonstrated improvements over conventional MRI, such as faster speed, noise reduction, and portability. Due to portability restrictions in several facilities with limited space, whenever possible, modular components that can be easily transported and assembled in different LMIC settings could improve flexibility, accessibility, and serviceability, particularly in mobile laboratories and remote areas. Furthermore, given the potential for LMFRI to revolutionize medical imaging in resource-constrained environments, educating parents and caregivers of children needing neuroimaging emerges as a crucial aspect. There is a need to share culturally appropriate materials to alleviate fears, enhance understanding, and maximize the benefits of caregiver involvement in supporting staff throughout the preparation and scanning process for optimal cooperation from the children and better-quality scans.

Variation in job roles and responsibilities, availability of human resources and basic infrastructure, clinic workflows, and imaging procedures across countries and between research sites affected the way users deal with challenges and interacted with the scanner, especially when positioning children for a scan. This reinforces the need for a human-centered and contextually appropriate approach to cater to the unique needs of LMIC populations. As such, user feedback, inclusive use case considerations, and a flexible and adaptive design and capacity-building philosophy would contribute to the development and implementation of scanners that maximize usability and accommodate the challenges and pressing health needs in LMICs. This could ultimately lead to increased access to and sustainable use of neuroimaging technology in diverse and resource-limited LMIC settings.

This study shows that LFMRI is intuitive and user-friendly, which is essential for widespread adoption, particularly in settings with diverse levels of technical expertise. Establishing continuous feedback mechanisms with end users allows for iterative improvements in technology and its application, drawing from real-world experiences to optimize effectiveness for local contexts. While LFMRI holds promise for improving neuroimaging access globally, priority areas for future research include the ease of use of data processing and scan interpretation, training on image quality to differentiate between noise and expected low resolution, and standardization of procedures and educational resources to enhance utility—all opportunities that could potentially be addressed through improvements and future software updates. In addition, policy and procurement factors that could significantly influence long-term LFMRI implementation and adoption, such as cost-effectiveness as well as budgeting for capital and ongoing operational and maintenance costs, potential upgrades, and workforce and national health strategy development, have not been fully explored. These considerations can support a better understanding of the benefits of low-field MRI as a valuable complementary tool to high-field MRI, thereby enhancing the overall capabilities of health systems and maximizing its impact on global health equity.

## Limitations

Data was self-reported and while all trained users were recruited, not all chose to participate, potentially limiting the robustness of data about site experiences. User insights from parents and caregivers of children who were scanned and the children themselves were not included in this study but could contribute information beneficial for decision-making regarding the development and implementation of LFMRI for pediatric neuroimaging.

## Conclusion

This usability and feasibility study of LFMRI reveals both challenges with and opportunities for enhancing pediatric imaging in resource-constrained settings from the perspectives of end users in LMICs. Users from all 11 sites were able to successfully operate LFMRI with in-person training and online support. The findings underscore the importance of design and training solutions that can be easily modified to accommodate diverse needs and workflow requirements across different populations and clinical applications. Addressing patient issues such as comfort, ventilation, and sensory experiences like noise, while prioritizing user-friendly interfaces and contextualizing training to the specific needs and backgrounds of the trainees is essential for sustainable, safe, and effective scanner usage. Moreover, infrastructure limitations, particularly regarding power instability and unreliable internet connectivity, prompts considerations for alternate power or offline strategies that could ensure functionality of the network infrastructure required for LFMRI operation, and potentially facilitate wider geographic accessibility in remote areas or clinics. This human-centered study focused on user experiences to provide insights for improving LFMRI and neuroimaging training, use, implementation, and education in LMICs. The study has uncovered user needs and challenges which play a crucial role in helping to improve the experience and quality of LFMRI use—some of which are already being implemented by UNITY consortium partners.

## Data Availability

The data that support the findings of the study are available from the corresponding authors upon reasonable request.

## Abbreviations

ICU, intensive care unit; LFMRI, low-field magnetic resonance imaging; LMICs, low- and middle-income countries; MRI, magnetic resonance imaging; UNITY, Ultra-low-field Neuroimaging In The Young.
